# Effectiveness of warm acupuncture and moxibustion in the treatment of osteoarthritis: a retrospective study on clinical and molecular outcomes

**DOI:** 10.3389/fmed.2026.1730151

**Published:** 2026-02-18

**Authors:** Jie Zhou, Kun Li, Yun Yang, Liangyu Lu

**Affiliations:** Department of Orthopedics, Pudong New Area People’s Hospital, Shanghai, China

**Keywords:** connexin, Cx43, inflammation, moxibustion, osteoarthritis, warm acupuncture

## Abstract

**Objectives:**

Osteoarthritis (OA) is a degenerative joint disease that causes pain and mobility loss in older adults. This study aimed to examine changes in connexin Cx26, Cx43, and Cx45 expression in OA patients after warm acupuncture with moxibustion.

**Methods:**

Records of 46 OA patients treated between March 2022 and December 2024 were reviewed, along with 46 age- and sex-matched healthy controls. This was a retrospective cohort study. Clinical scores [Lysholm knee score, visual analog scale (WOMAC)] were used to assess joint function, pain, and mobility. Warm acupuncture with moxibustion was applied following a standardized protocol, targeting specific acupoints, including Dubi (ST35), Zusanli (ST36), Yinlingquan (SP9), Yanglingquan (GB34), and Heding. The intervention was administered three times per week for 8 weeks. Laboratory data on bone metabolism markers [osteoprotegerin (OPG), and bone gla protein (BGP)], inflammatory mediators [MMP-1, MMP-3, prostaglandin E2, interleukin-1β, and tumor necrosis factor-α (TNF-α)], and the mRNA expression of Cx26, Cx43, and Cx45 were analyzed.

**Results:**

Post-treatment, the Lysholm knee score (LKSS) increased and the VAS and WOMAC scores decreased (*P* < 0.05). OPG and BGP levels increased, whereas MMP-1, MMP-3, PGE2, IL-1β, and TNF-α levels decreased (*P* < 0.05). Cx26 expression decreased, whereas Cx43 and Cx45 expression increased (*P* < 0.05). Connexin levels post-treatment were comparable to those of the controls (*P* > 0.05).

**Conclusions:**

Warm acupuncture with moxibustion improved joint function, reduced pain and inflammation, and normalized connexin expression in OA patients. The standardized treatment protocol and the retrospective cohort design provide insight into the potential clinical benefits of this approach for OA management.

## 1 Background

Osteoarthritis (OA) is a mild inflammatory disease of the synovial joint and the most common form of arthritis. As a result, elderly people suffer chronic pain and disabilities related to their limbs (1, 2). In the past, OA was thought to be caused by decades of wear; however, currently, it is believed that OA is an inflammatory, biomechanical organ disease associated with systemic disease. The factors affecting the occurrence and development of OA include innate immunity, complement proteins, inflammatory mediators, bone form, osteoarticular dysplasia, obesity, and mild inflammation caused by systemic metabolic diseases (3). The progressive degeneration of the articular cartilage matrix is the main cause of pain and disability.

In cartilage, although chondrocytes make up only 10% of the volume of articular cartilage, they are important structures for maintaining the anabolic balance of articular cartilage (4). Each chondrocyte exists individually within the cartilage cavity, a feature that allows no direct contact between chondrocytes, which are involved primarily in intercellular signaling through the diffusion of material from the matrix. However, for articular cartilage to respond promptly and uniformly to biological stimuli, injury, or simple maintenance of homeostasis, proper intercellular communication is critical (5). At present, a majority of OA patients are treated symptomatically.

Chinese medicine has also achieved safe and good clinical efficacy in the prevention and management of OA. Chinese medicine has the advantages of simple operation, safety, and economy, and there are many treatment options. Among the many Chinese medical treatments, warm acupuncture has been widely used in recent years to treat osteoarthritis of the knee (KOA) because of its ability to warm meridians, move Qi and activate blood (6). The name warm acupuncture was first described in the Treatise on febrile Diseases, but there were no detailed operational records. Acupuncture Juying in Ming Gaowu and Dacheng in Yang Jizhou were recorded as follows: on acupuncture points, with Angelica dahurica da. Warm acupuncture is especially suitable for the symptoms of cold and dampness and meridian stagnation, such as arthralgia, epigastric pain, abdominal pain and diarrhea (7, 8).

In the context of OA, various biomarkers play a crucial role in the pathophysiology, particularly in inflammation and cartilage degradation. Inflammatory mediators such as MMP-1, MMP-3, PGE2, IL-1β, and TNF-α are central to the catabolic processes of cartilage breakdown. MMPs degrade the extracellular matrix, while PGE2 and inflammatory cytokines such as IL-1β and TNF-α amplify the inflammatory response, contributing to the pain and stiffness characteristic of OA. Bone metabolism markers, including OPG and BGP, are involved in the regulation of bone resorption and formation, processes that are often disrupted in OA. Elevated OPG levels inhibit osteoclast activity, whereas BGP helps maintain bone integrity.

Connexins (Cx26, Cx43, and Cx45) are proteins that form gap junctions, facilitating intercellular communication, and are crucial in maintaining cartilage homeostasis. Cx43 and Cx45 regulate chondrocyte function and matrix production, while Cx26 plays a role in ion transport, which is essential for cellular integrity. Dysregulation of connexin expression contributes to cartilage degradation and the progression of OA. Although previous research has documented the role of inflammatory mediators and bone metabolism markers in OA, no studies have yet explored the changes in connexin expression (Cx26, Cx43, Cx45) in OA patients before and after treatment. This study aims to fill that gap by investigating the potential relationship between warm acupuncture with moxibustion treatment and the normalization of connexin expression in OA patients. We hypothesize that warm acupuncture may modulate these biomarkers, particularly by reducing inflammation and promoting bone remodeling, thus improving joint function and reducing pain.

Understanding these mechanisms and how acupuncture influences biomarker expression is crucial for identifying effective treatments for knee OA, a leading cause of disability worldwide, especially among elderly individuals. Improving the treatment of OA will enhance patients’ quality of life and potentially reduce the societal burden of this disease.

## 2 Patients and methods

### 2.1 General information

Between March 2022 and December 2024, the medical records of 46 patients who were diagnosed with OA and treated at our hospital with warm acupuncture and moxibustion for 8 weeks were retrospectively reviewed and designated the study group (SG). In addition, 46 healthy adults who underwent routine physical examinations at the same hospital during the same period composed the control group (CG), with no history of OA or relevant intervention. The study followed a retrospective cohort design, and both groups were matched for age, sex, and other relevant variables.

The sample size of 46 patients in each group was not determined by an a priori sample size calculation, as this was a retrospective study utilizing available patient records. However, we included all eligible patients who met the inclusion criteria within the study period. Given the relatively small sample size, the findings should be interpreted with caution. Additionally, patients were selected based on consecutive enrollment, where eligible patients from the hospital’s records were included as they presented with OA during the study period. Matching was performed for age, sex, and other relevant variables, although random sampling was not used due to the retrospective design of the study.

The SG included 17 women and 29 men, with a mean age of 59.28 ± 6.61 years. The average disease duration was 4.52 ± 0.6 years (range: 1–7 years) (Table 1). The CG comprised 20 women and 26 men, with a mean age of 59.10 ± 6.55 years. There were no statistically significant differences between the two groups in terms of age, sex distribution, body mass index, or educational level (*P* > 0.05).

**TABLE 1:** Baseline characteristics of study participants.

Characteristic	Study group (SG)	Control group (CG)
Age (years)	59.28 ± 6.61	59.10 ± 6.55
Sex (Female:Male)	17:29	20:26
Disease duration (years)	4.52 ± 0.6	–
Kellgren–Lawrence Grading	2–3 (Moderate OA)	–
Imaging findings	Joint space narrowing, osteophytes, subchondral sclerosis	–
Affected side (right/left)	24 Right/22 Left	–
Presence of synovitis	40%	–
Presence of osteophytes	85%	–
Other OA severity indicators	Pain, swelling, reduced range of motion	–

The values are expressed as the means ± standard deviations or as percentages where appropriate. The study group (SG) consisted of osteoarthritis (OA) patients treated with warm acupuncture and moxibustion, whereas the control group (CG) consisted of healthy individuals with no history of OA. Disease duration refers to the length of time the patients have experienced OA. The Kellgren–Lawrence grading reflects the severity of OA on the basis of radiographic findings. Imaging findings include joint space narrowing, osteophytes, and subchondral sclerosis. The presence of synovitis and osteophytes was assessed as indicators of OA severity.

The study protocol was reviewed and approved by the Ethics Committee of our hospital (approval number: PPH202503101). All the data used in this study were anonymized and handled in compliance with institutional and national ethical standards.

The diagnostic criteria of the SG are as follows: (1) the standard of traditional Chinese medicine (TCM) refers to the diagnostic criteria of bone arthralgia syndrome according to the diagnostic and curative effect standard of TCM disease syndrome (9): the dialectical type is Yang deficiency and cold coagulation type, the main syndrome is unfavorable flexion and extension, limb joint pain, and the secondary syndrome is clanging white or swarthy complexion. The limbs were not warm, and the whole body was afraid of cold, fatigue, white coating, light tongue, and slow pulse. (2) Western medical standards refer to the following guidelines for diagnosing and treating OA (10): (1) white blood cell count < 2,000/mL, with the articular fluid (at least twice) clear and sticky; (2) X-ray showing joint lesions; 3) recurrent knee joint pain (within 1 month); (4) stiffness in the morning for ≤ 30 min; and (5) a sound of bone friction during activity.

The inclusion criteria were as follows: (1) a patient must meet the above diagnostic criteria and not receive any other treatment in the last 4 weeks to be considered for the study; (2) the clinical data were complete; and (3) as this study was retrospective, the informed consent process was waived by the Ethics Committee because of the use of anonymized data from medical records. However, all participants’ data were handled in compliance with institutional and national ethical standards; (4) the study included patients diagnosed with osteoarthritis (OA) who were treated with warm acupuncture and moxibustion. Patients with moderate OA (grades 2–3) according to the Kellgren–Lawrence classification were included in the study. The participants presented symptoms of joint pain, stiffness, and limitations in joint movement. These patients were selected based on radiographic findings of joint space narrowing, osteophytes, and subchondral sclerosis. The study group consisted of 46 patients, and they were all evaluated for their clinical and laboratory data before and after treatment. The exclusion criteria were as follows: (1) individuals with other painful diseases of the knee joint; (2) individuals with mental illness who were unable to collaborate with the study; (3) patients who had received related drug treatment in the past month; (4) patients with malignant tumors; (5) special groups such as pregnant and lactating women; and (6) diabetic patients were excluded due to the potential risk of complications such as impaired wound healing and skin sensitivity, particularly in those receiving moxibustion therapy.

To prevent the risk of burns, especially in individuals with skin sensitivity, precautions were taken during the moxibustion treatments. The skin temperature was closely monitored, and the moxa sticks were kept at a safe distance from the skin. Treatment times were adjusted based on individual patient tolerance, and patients were regularly assessed for signs of skin irritation or burns during the sessions. Additionally, patients with any history of skin sensitivity were excluded from the study to minimize the risk of burns.

### 2.2 RT-qPCR experiment for connexin expression

The expression levels of connexins Cx26, Cx43, and Cx45 were analyzed using reverse transcription quantitative polymerase chain reaction (RT-qPCR). Articular cartilage samples were obtained from OA patients undergoing knee joint surgery at our hospital, specifically from the femoral condyle and tibial plateau. These samples were immediately stored in liquid nitrogen, thawed, minced, and homogenized in RNA extraction buffer. Total RNA was extracted using TRIzol reagent (Invitrogen, United States), and the RNA concentration and purity were determined using a NanoDrop spectrophotometer, with only samples having an A260/A280 ratio between 1.8 and 2.0 used for further analysis. cDNA was synthesized using the PrimeScript RT Reagent Kit (TaKaRa, Japan), following the manufacturer’s protocol. The primers for Cx26, Cx43, Cx45, and GAPDH were designed using Primer3 software, and the amplification conditions were set as follows: initial denaturation at 95°C for 5 min, followed by 40 cycles of denaturation at 95°C for 30 s, annealing at 60°C for 30 s, and extension at 72°C for 30 s. Melting curve analysis was conducted to ensure the specificity of the amplification. GAPDH was used as the internal reference gene due to its stable expression across OA tissue types. The relative expression levels of Cx26, Cx43, and Cx45 were calculated using the 2-ΔΔCt method.

### 2.3 Treatment methods

Patients in the study group (SG) had previously received warm acupuncture combined with moxibustion, as documented in their medical records. A Yunlong acupuncture needle (0.30 mm × 40 mm) was used. With the affected knee in a naturally extended position, patients were placed in the supine posture. Following skin disinfection, acupuncture was applied to points around the knee joint, including Dubi (ST35), Zusanli (ST36), Yinlingquan (SP9), Yanglingquan (GB34), and Heding. The Neiguan and Dubi points were punctured obliquely at approximately 2.5 cm upward at a 45° angle. The needling technique involves tonifying, diaphoresis, and descending qi methods. For warm moxibustion, a 2 cm section of moxa stick was affixed to the needle handle. Two consecutive sections of moxa were burned for each session before needle withdrawal. Treatments were administered three times per week over an 8-week course.

### 2.4 Observation index

(1) The clinical efficacy in individuals in the SG was evaluated after 8 weeks of treatment. The criteria for judging the curative effect were as follows: the Diagnostic Efficacy Standard of Diseases and Syndromes of TCM (9). The main symptoms included knee joint soreness or distension or tingling, unfavorable flexion and extension, aggravation of cold symptoms and relief of fever symptoms. The cold sensation of the limbs and the severity of day and night were judged by personnel who did not participate in this study. The cure was the main syndrome of the patient, the concurrent syndrome disappeared, and the activity function returned to its typical state. Effective rate = (cured cases + markedly effective cases + effective cases)/observed cases × 100%. (2) The Lysholm knee joint function rating scale (LKSS) (11) was employed to assess the knee joint function of individuals in the SG, including claudication, joint instability, pain, support, dyskinesia, noose, and swelling, with a total score of 100. (3) The pain visual analogue scale (VAS) (12) was adopted to assess the degree of pain experienced by individuals in the SG. The VAS score was divided into 10 scales, with 0 indicating no pain and 10 indicating unbearable severe pain. (4) The knee joint health status of individuals in the SG including pain, stiffness, difficulty performing daily activities and other dimensions, was evaluated via the Xi’an OA Index (WOM-WOMAC) and McMaster University OA Index (OA Index) (13). In the form of a questionnaire, each item was scored as 0: 4 according to severity, with the highest score being 96. The patients were evaluated before treatment and 8 weeks after treatment. (5) Referring to the Guidelines for Clinical Research on New Chinese Medicines (14), the patients in the SG had changes in symptom scores before and after treatment: joint pain, joint flexion and extension, joint pressure, swelling of the lower limbs, joint skin fever, walking pain and discomfort. Based on the patients’ clinical presentation, each clinical symptom was categorized as none (0 points), mild (2 points), moderate (4 points), and severe (6 points). (6) For the detection of bone metabolic indices in the SG 4–7 mL of venous blood was extracted from each group before and 8 weeks after therapy. The serum was obtained after centrifugation in the laboratory department of our hospital. The levels of calcitonin (CT), OPG and osteocalcin (BGP) were detected by radioimmunoassay. The kits were acquired from Nanjing Sailui Biotechnology Co., Ltd., and Shanghai Yubo Biotechnology Co., Ltd. (7) Before and 8 weeks after treatment, the serum factors of the SG, as well as the fasting venous blood volume (4 ml) from each patient were determined. After being incubated at 3,000 r/min for 10 min (centrifugal radius of 10 cm), the upper layer of serum was collected. In addition, prostaglandin E2 (PGE2), interleukin-1 β (IL-1β), tumor necrosis factor-α (TNF-α) and matrix metalloproteinases (MMPs) were detected by ELISA. The MMP-1 and MMP-3 assays were carried out in strict accordance with the instructions of the kits, which were acquired from Shanghai Senxiong Biological Co., Ltd. (8) The expression of the gap junction proteins Cx26, Cx43, and CX45 in human articular chondrocytes was detected by RT-qPCR. The patients were evaluated before treatment and 8 weeks after treatment.

### 2.5 Statistical analysis

The data were analyzed using SPSS 20.0 (IBM, United States). The normality of the data was assessed using the Shapiro-Wilk test for all continuous variables. For variables with a normal distribution, parametric tests were applied, including paired *t*-tests for within-group comparisons and independent *t*-tests for between-group comparisons. For non-normally distributed data, non-parametric tests, such as the Wilcoxon signed-rank test, were applied for paired comparisons, and the Mann-Whitney U test was used for comparisons between the SG and the CG. The Chi-square test was employed for categorical data, including sex distribution and other frequency-based comparisons. For multiple comparisons, the Bonferroni correction was applied to adjust for the increased risk of Type I error. Exact *p*-values and 95% confidence intervals (CIs) were reported for all relevant analyses. A *p*-value of < 0.05 was considered statistically significant. Additionally, to assess the magnitude of the effects, we calculated effect sizes for the statistical tests, including Cohen’s d for t-tests and r for correlation coefficients. This was done to provide additional insight into the practical significance of the observed results.

## 3 Results

### 3.1 Therapeutic effect of 46 patients with OA

**FIGURE 1:**
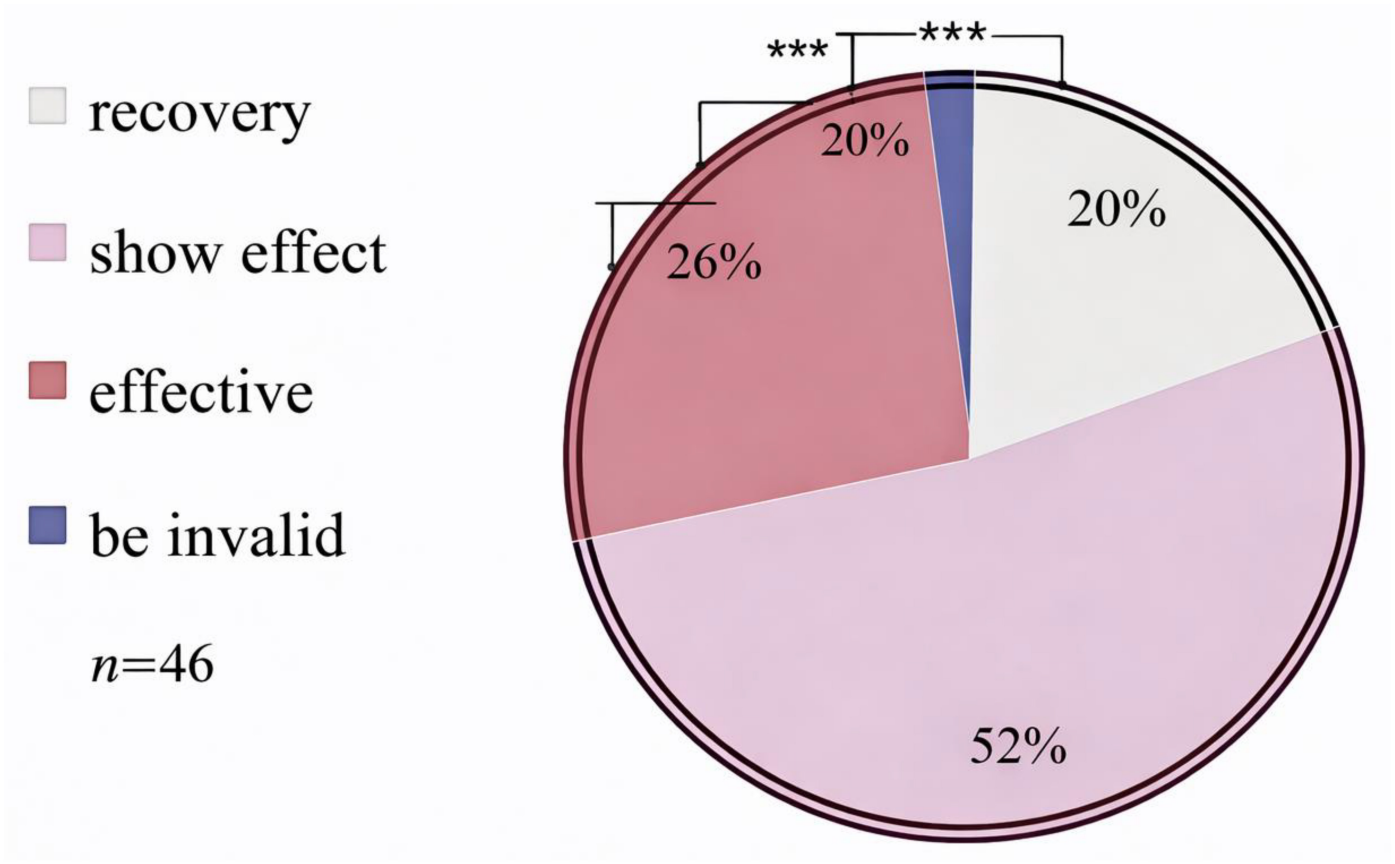
Therapeutic effects in 46 patients. This pie chart illustrates the therapeutic effects in osteoarthritis (OA) patients after treatment, categorized into recovery, effective, and invalid effects. The sample size (*n* = 46) is indicated, and the error bars represent confidence intervals. ***Indicates *P* < 0.001.

After treatment, 9 patients were cured, 24 patients were noticeably effective, 12 patients were effective, 1 patient was ineffective, and the effective rate was 97.83%. All the results are shown in Figure 1.

### 3.2 Comparison of the Lysholm score, VAS score and WOMAC score in patients with OA before and after treatment

The Lysholm score of patients with OA after treatment was noticeably higher than that before treatment. In addition, the VSA score and WOMAC score were noticeably lower than those before treatment (*P* < 0.05). Table 2 displays all of the findings.

**TABLE 2:** Lysholm score, VAS score and WOMAC score in patients with OA before and after treatment ($\bar{x}$ ± s, points).

Group	N	Lysholm scoring	VSA scoring	WOMAC scoring
Before treatment	46	47.43 ± 4.61	2.97 ± 0.68	37.58 ± 5.07
After treatment	*46*	81.32 ± 11.07	0.35 ± 0.51	7.09 ± 2.57
*T*		19.168	20.906	36.801
*P*		<0.05	<0.05	<0.05

### 3.3 Clinical symptom scores in patients with OA before and after treatment

After treatment, the scores of clinical symptoms such as joint pain, joint flexion and extension, joint tenderness, lower limb swelling, joint skin fever walking pain and discomfort in patients with OA were obviously lower than those before treatment (*P* < 0.05), as shown in Table 3.

**TABLE 3:** Clinical symptom scores of patients with OA before and after treatment ($\bar{x}$ ± s, points).

Group	N	Joint pain	Poor flexion and extension of joint	Joint tenderness	Acid distension of lower limbs	Joint skin fever	Walking pain and discomfort
Before treatment	46	3.67 ± 1.04	4.03 ± 0.76	3.71 ± 0.58	2.89 ± 0.78	2.71 ± 0.66	4.29 ± 0.71
After treatment	46	1.47 ± 0.45	1.36 ± 0.57	1.07 ± 0.48	0.87 ± 0.64	0.73 ± 0.53	1.43 ± 0.56
*t*		13.167	19.062	23.783	13.579 <0.05	15.865 <0.05	21.451 <0.05
*P*		<0.05	<0.05	<0.05			

### 3.4 Bone metabolic indices in patients with OA before and after treatment

There was no noticeable change in CT levels in patients with OA before and after treatment (*P* > 0.05). The levels of OPG and BGP in patients with OA were noticeably increased (*P* < 0.05). Table 4 displays all of the findings.

**TABLE 4:** Bone metabolic indexes in patients with OA before and after treatment ($\bar{x}$ ± s).

Group	N	OPG (ng/L)	CT (ng/L)	BGP (mg/L)
Before treatment	46	3.67 ± 0.35	10.54 ± 0.41	3.34 ± 0.38
After treatment	46	4.88 ± 0.29	10.62 ± 0.53	6.31 ± 0.57
*T*		18.055	0.810	29.404
*P*		<0.05	>0.05	<0.05

### 3.5 Serum indices in patients with OA before and after treatment

Compared with those before treatment, the serum levels of MMP-1, MMP-3, PGE2, IL-1β, and TNF-α in patients with OA after treatment were noticeably decreased (*P* < 0.05) (Table 5).

**TABLE 5:** Serum indices in individuals with OA before and after treatment ($\bar{x}$ ± s).

Group	N	MMP-1 (μg/L)	MMP-3 (μg/L)	PGE_2_ (pg/mL)	IL-1β (pg/mL)	TNF-α (pg/mL)
Before treatment	46	0.56 ± 0.08	25.91 ± 6.87	1.35 ± 0.33	6.15 ± 1.13	9.84 ± 1.26
After treatment	46	0.34 ± 0.07	13.25 ± 4.26	0.41 ± 0.07	2.34 ± 0.25	5.22 ± 0.64
*T*		14.037	10.622	18.899	22.328	22.172
*P*		<0.05	<0.05	<0.05	<0.05	<0.05

### 3.6 The expression levels of the gap junction proteins Cx26, Cx43, and CX45

**FIGURE 2:**
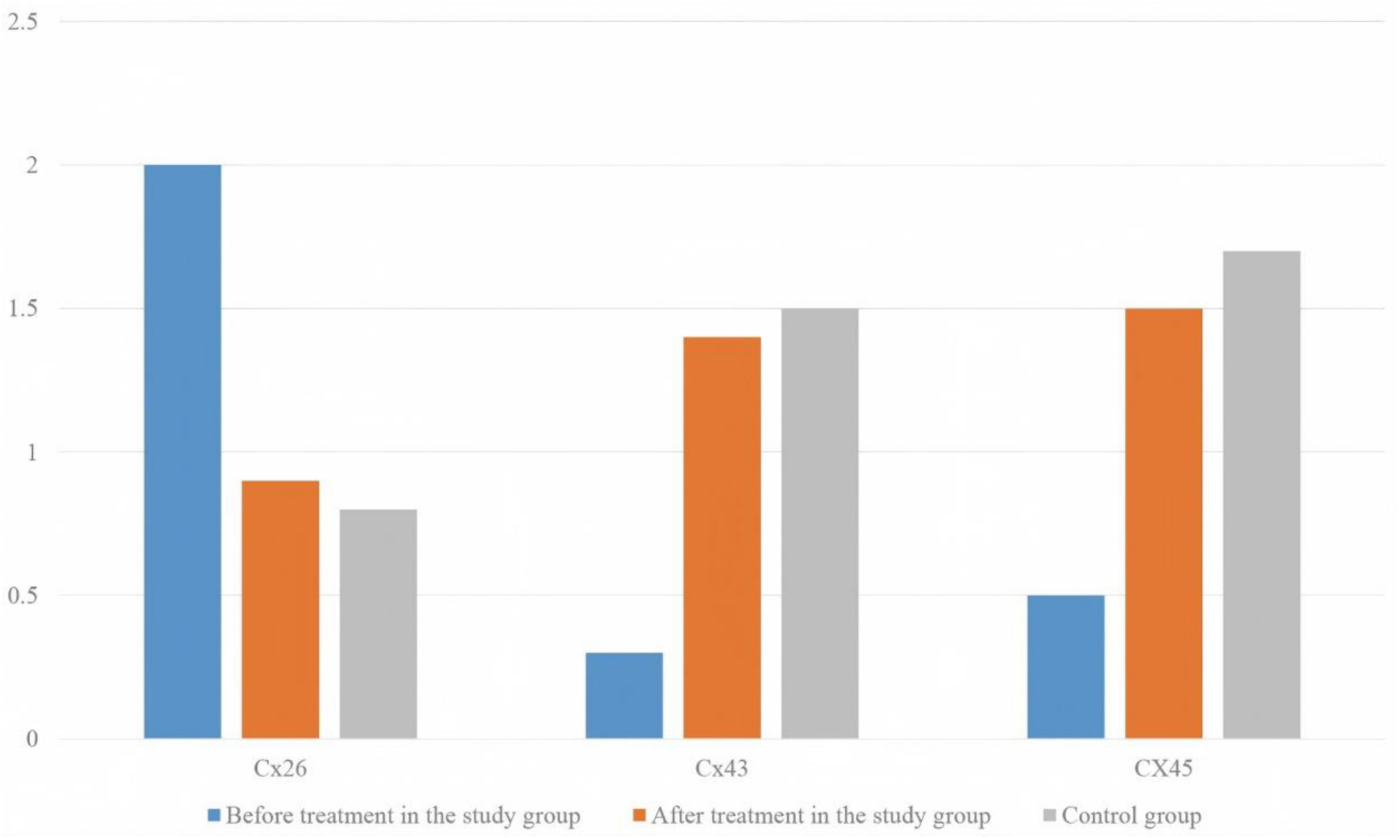
Cx26, Cx43, and CX45 expression levels in patients with OA before and after treatment.

The levels of connexin Cx26 mRNA in the SGs after treatment were noticeably lower than those before treatment. The levels of Cx43 mRNA and CX45 mRNA were noticeably higher than those before treatment (*P* < 0.05). Before treatment, the level of Cx26 mRNA in the SG was higher, and the levels of Cx43 mRNA and CX45 mRNA were lower (*P* < 0.05). After treatment, the levels of Cx26 mRNA, Cx43 mRNA and CX45 mRNA in the SG were close to those in the CG (*P* > 0.05) (Figure 2).

## 4 Discussion

OA is a type of degenerative bone disease, that is characterized by progressive subchondral bone regeneration and bone defects. Intra-articular cartilage cystic degeneration, hardening and fracture hyperplasia lead to narrowing of the joint space, resulting in joint swelling, pain, and mobility disorders. OA is caused by an imbalance between extracellular matrix degradation and chondrocyte synthesis, resulting in mechanical and biological factors working together. The pathogenic factors of OA are complicated. It is related mainly to endocrine system disorders, obesity, genetic factors, and age (15). The initial onset is joint pain, which can develop into persistent joint pain with limited joint movement and even limping (16). As OA is difficult to detect in its early stages, taking effective measures to control the progression of the disease in a timely manner can contribute favorably to lowering the suffering of patients and improving their prognosis.

At present, Western medicine commonly treats OA using non-steroidal anti-inflammatory drugs, intra-articular sodium hyaluronate injections, artificial joints, and knee debridement. Its advantage is that the short-term effect is rapid, but the long-term effect cannot be guaranteed. Oral non-steroidal anti-inflammatory drugs take effect quickly, but there are some problems with long-term oral administration, such as drug dependence, drug resistance, gastrointestinal stimulation, and liver, and kidney side effects. In contrast, surgical treatment is invasive and can lead to many complications, such as post-operative infections. TCM has a long history of using acupuncture to treat bone arthralgia. In accordance with the characteristics of meridian circulation and acupuncture point treatment, stimulation of meridians and acupuncture points harmonizes yin and yang, liaises with internal organs, runs qi and blood, communicates internally and externally, and regulates the body’s activities. At the same time, we utilized complementary and diaphoretic techniques to stimulate the corresponding acupuncture points to unblock the meridians, harmonize yin and yang, and tonify deficiency and dispel evil. “Jade Dragon Song” records that the knees and legs are incapable of standing up as a man. Acupuncture can better eliminate local soft tissue spasms, promote blood circulation, improve local metabolism, inhibit or eliminate the production of local inflammatory substances, inhibit pain centers in the cerebral cortex, block the conduction of central nervous system receptors and enhance the analgesic effect. Acupuncture and moxibustion therapy are widely used in clinical practice because of their advantages of simple operation, obvious effects, and no obvious adverse effects (17, 18). The combination of acupuncture and moxibustion can nourish the kidney and liver, loosen adhesions, restore the elasticity of muscles and tendons, increase the range of motion of joints, relieve spasm and pain, release adhesions, and smooth joints, and improve local blood circulation. In addition, acupuncture at the corresponding points improves ischemia, promotes the absorption of stagnant blood and reduces pressure on the knee joint, thereby restoring the balance of pressure, stress and tension in the knee joint.

The current findings may indicate improvement in OA, and the treatment efficiency was as high as 97.83%. The scores of clinical symptoms such as swelling, joint skin fever, walking pain and discomfort were noticeably decreased. The clinical symptom scores of OA individuals noticeably reduced after treatment, such as joint pain, unfavorable joint flexion and extension, joint tenderness, lower extremity soreness, joint skin fever, walking pain and discomfort. This suggests that warm acupuncture may be associated with the improvement of clinical symptoms, which could help reduce the intensity of discomfort and support knee joint recuperation. The clinical symptoms of patients have been noticeably improved, which could contribute to reducing their pain and make them return to normal life as soon as possible. From a long-term point of view, this may help enhance the quality of life of individuals. Moreover, these findings fully support the scientific nature and effectiveness of this treatment. In addition, abnormal changes of bone metabolic indices are important for the pathogenesis of OA, in which CT can reflect the level in bone resorption and osteoclasts play a crucial role in the process of bone resorption, whereas BGP and OPG can reflect the level of bone formation and effectively inhibit the proliferation and growth of osteoclasts (19). The findings of this study suggest that the levels of OPG and BGP in patients with OA after treatment were noticeably higher than those before treatment. These findings indicate that warm acupuncture may be associated with improvements in the bone metabolism indices of OA patients and promote disease recovery. Among the acupuncture points selected for warm acupuncture, the inner knee eye point is a special point outside the meridian, that is mainly used to treat pain in the middle of the knee (20–22). The Zusanli acupoint is the acupoint of Yangming’s stomach meridian, which is used for strengthening the spleen, tonifying qi, dispelling wind, and dredging collaterals. The Dubi acupoint dredges meridians, activates collaterals, regulates qi and relieves pain. Heding acupoints, which are special acupoints of the lower limbs are experience points for the treatment of knee joint pain. Yinlingquan and Yanglingquan are the main acupoints for the treatment of meridian and tendon lesions and joint pain, which are beneficial for qi and blood. The above-mentioned acupoints were performed together to invigorate the deficiency, clear the collaterals, consolidate the root, and dispel pathogens. In addition, warm acupuncture and moxibustion can make warm power and acupuncture feel directly to the affected area through the filiform needle, promote the absorption of blood stasis, facilitate the absorption of inflammation in the body, relieve the patient’s joint pain and local muscle spasm, and help the patient’s subsequent joint recovery.

Several studies have investigated the efficacy of different therapeutic modalities for treating osteoarthritis, including extracorporeal shock wave therapy and low-level laser therapy. For example, Öztürk and Yetişir (23) explored the effects of extracorporeal shock wave therapy in calcaneal spurs, while Yetişir and Öztürk (24) assessed the effects of low-level laser therapy on knee pain and function in patients with knee osteoarthritis (23, 24). These studies suggest that non-invasive therapies can significantly reduce pain and improve joint function, which aligns with the findings from our study on the effectiveness of warm acupuncture and moxibustion.

Gap junctions are transmembrane channels located on adjacent cell membranes, allowing molecules with a relative molecular weight of less than 1,000 or less than 1.5 nm, such as cyclic adenosine monophosphate (cAMP), Ca^2+^ inositol triphosphate (inositol), and adenosine triphosphate (ATP), to pass through. It is the most common connection between cells and the main material basis for the transmission of electrical activity between cells. It is closely related to the regulation of cell growth, differentiation, and proliferation. The types of gap junction proteins include Cx26, Cx30, Cx31, Cx32, Cx33, Cx37, Cx40, Cx43, Cx45, Cx46, Cx50, and Cx57 (25, 26). The increased intercellular communication of gap junction channels has been confirmed to be related to the secretion of the MMP (27, 28). In synovial cells treated with junction channel inhibitors, the MMP induced by IL-1β decreased noticeably. The destruction of articular cartilage by decomposing factors such as the MMP depends on the existence of Cx43 channels (29). Among them, the half channel formed by Cx43 is an important pathway for the release of PGE2 and ATP outside the cell matrix. Increased expression of connexin can increase the expression levels of MMP-1 and MMP-13, which can enhance the secretion of collagenase in cell culture medium. In addition, the study revealed that connex is strongly associated with the formation of an inflammatory state. In the nervous system, the expression of silent connexin genes can noticeably down-regulate the inflammatory response (30). In addition, in the rat model of rheumatoid arthritis, the expression of silent connexin noticeably reduced the expression of inflammatory factors and joint injury was noticeably improved (31). The close relationship between ligand proteins, inflammatory cytokines and the MMP allows ligand proteins to play a role in regulating the osteoarthritic microenvironment. In this study, the levels of serum MMP-1, MMP-3, PGE2, IL-1β, and TNF-α in the SGs after treatment were noticeably lower than those before treatment, which was consistent with the previous analysis. Additionally, the findings of the present study demonstrated that compared with those before treatment, the connexin Cx26 mRNA in the SGs decreased after treatment, and the levels of Cx43 mRNA and CX45 mRNA increased. These findings suggest that warm acupuncture can inhibit the inflammatory response of the body, which may help minimize the level of inflammatory factors in patients. The expression levels of connexin Cx26, Cx43, and CX45 can be adopted as reference indicators to evaluate the clinical efficacy of treatment in patients with OA. After that, the level of Cx26 mRNA is greatly reduced, and the levels of Cx43 mRNA and CX45 mRNA are noticeably increased. Before treatment, the level of Cx26 mRNA in the SG was higher, and the levels of Cx43 and CX45 mRNAs were lower. After treatment, the levels of Cx26, Cx43, and CX45 mRNAs in the SG were close to those in the CG. The results revealed that the levels of Cx26, Cx43 and CX45 mRNAs in the SGs gradually returned to normal after treatment. Furthermore, that there was a certain correlation between the expression levels of Cx26, Cx43, and CX45 mRNAs and patient condition.

## 5 Limitations

This study has several limitations that should be considered when interpreting the results. First, the single-center design restricts the generalizability of our findings. Second, the small sample size of 46 participants may limit the statistical power and precision of the results. Additionally, the retrospective nature of the study introduces the potential for bias and confounding variables, as we did not control for all factors that could influence the outcomes. Third, the short follow-up duration did not allow us to assess the long-term efficacy and safety of warm acupuncture with moxibustion. Fourth, the study did not include imaging outcomes such as radiographic or MRI assessments, which could provide more objective measures of joint damage or improvement. Finally, the lack of blinding in the study may have introduced bias in the evaluation of clinical outcomes, as both the patients and healthcare providers were aware of the treatment being administered.

## 6 Conclusion

In summary, warm acupuncture can improve the joint function of patients with OA, reduce the degree of pain, inhibit the expression of inflammatory factors, and regulate the expression of the gap junction proteins Cx26, Cx43, and CX45. A large sample clinical study with a scientific design, rigorous implementation and reliable results is still needed.

## Data Availability

The raw data supporting the conclusions of this article will be made available by the authors, without undue reservation.
